# Factors Affecting Patients with Concurrent Deep Neck Infection and Acute Epiglottitis

**DOI:** 10.3390/diagnostics12010029

**Published:** 2021-12-23

**Authors:** Chia-Ying Ho, Yu-Chien Wang, Shy-Chyi Chin, Shih-Lung Chen

**Affiliations:** 1 Center for Traditional Chinese Medicine, Division of Chinese Internal Medicine, Chang Gung Memorial Hospital, Taoyuan 333, Taiwan; chiayingho23@gmail.com; 2 School of Medicine, Chang Gung University, Taoyuan 333, Taiwan; m7054@cgmh.org.tw (Y.-C.W.); b25chin@gmail.com (S.-C.C.); 3 Department of Otorhinolaryngology & Head and Neck Surgery, New Taipei Municipal TuCheng Hospital, New Taipei City 236, Taiwan; 4 Department of Otorhinolaryngology & Head and Neck Surgery, Chang Gung Memorial Hospital, Linkou 333, Taiwan; 5 Department of Medical Imaging and Intervention, Chang Gung Memorial Hospital, Linkou 333, Taiwan

**Keywords:** deep neck infection, acute epiglottitis, parapharyngeal space, submandibular space

## Abstract

Deep neck infection (DNI) is a serious disease of deep neck spaces that can lead to morbidities and mortality. Acute epiglottitis (AE) is a severe infection of the epiglottis, which can lead to airway obstruction. However, there have been no studies of risk factors in patients with concurrent DNI and AE. This study was performed to investigate this issue. A total of 502 subjects with DNI were enrolled in the study between June 2016 and August 2021. Among these patients, 30 had concurrent DNI and AE. The relevant clinical variables were assessed. In a univariate analysis, involvement of the parapharyngeal space (OR = 21.50, 95% CI: 2.905–158.7, *p* < 0.001) and involvement of the submandibular space (OR = 2.064, 95% CI: 0.961–4.434, *p* < 0.001) were significant risk factors for concurrent DNI and AE. In a multivariate analysis, involvement of the parapharyngeal space (OR = 23.69, 95% CI: 3.187–175.4, *p* = 0.002) and involvement of the submandibular space (OR = 2.465, 95% CI: 1.131–5.375, *p* < 0.023) were independent risk factors for patients with concurrent DNI and AE. There were no differences in pathogens, therapeutic managements (tracheostomy, intubation, surgical drainage), or hospital staying period between the 30 patients with concurrent DNI and AE and the 472 patients with DNI alone (all *p* > 0.05). However, we believe it is significant that DNI and AE are concurrent because both DNI and AE potentially cause airway obstruction, and concurrence of these two diseases make airway protection more difficult. The infections in critical spaces may cause the coincidence of these two diseases. Involvement of the parapharyngeal space and involvement of the submandibular space were independent risk factors associated with concurrent DNI and AE. There were no differences in pathogens between the concurrent DNI and AE group and the DNI alone group.

## 1. Introduction

Deep neck infection (DNI) is a lethal bacterial infection in the potential spaces of the neck [1]. DNI can lead to airway obstruction and cause severe morbidity and mortality, including severe sepsis, esophageal perforation, necrotizing fasciitis, descending necrotizing mediastinitis, disseminated intravascular coagulation, jugular vein thrombosis, and even pericarditis [2,3,4,5,6].

Acute epiglottitis (AE) is a bacterial infection of supraglottic structures that results in a symptom complex consisting of a sore throat, stridor, odynophagia, muffled voice, and a high fever; it is a potentially life-threatening condition secondary to airway obstruction [7]. 

DNI is clinically first suspected in patients with shortness of breath, local heat, redness, and swelling in the neck. The otolaryngologist will then use a flexible fiberoptic laryngoscope to look for an upper airway obstruction, and the coexistence of AE may be found.

Previous studies showed that some patients may present with concurrent DNI and AE [8,9]. Therapeutic management in patients with the cooccurrence of these two lethal diseases is complicated. AE necessitates protection of the airway, and severe DNI can cause septic shock and comorbidity. However, there have been no previous studies regarding risk factors for the cooccurrence of these two diseases. This study was therefore performed to investigate the risk factors in patients with concurrent DNI and AE.

## 2. Materials and Methods

**Figure 1:**
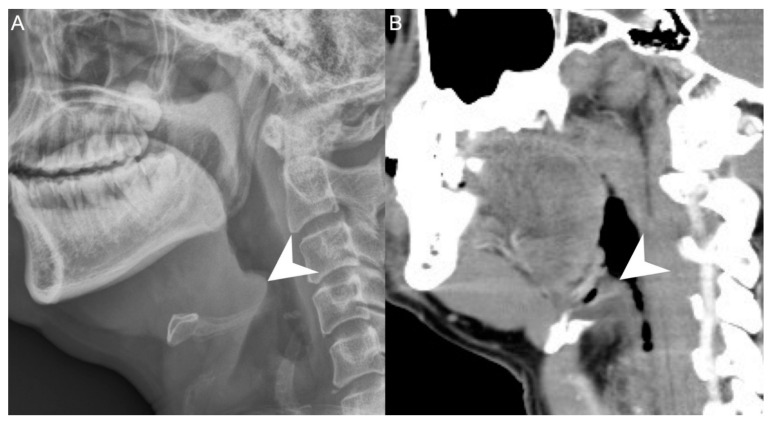
A patient with concurrent acute epiglottitis and deep neck infection as seen from a (**A**) neck lateral view and (**B**) sagittal view on CT. Arrowhead: swelling of the epiglottis. 300 × 300 DPI.

We retrospectively reviewed the medical records of 502 patients diagnosed with DNI who were admitted to Chang Gung Memorial Hospital in Linkou, Taiwan, between June 2016 and August 2021. DNI diagnosis was performed by ultrasonography (USA) and computed tomography (CT), while AE was diagnosed by a lateral neck X-ray (Figure 1), flexible fiberoptic laryngoscopy, and CT. The treatment course included antibiotics, US-guided needle drainage, and open surgical incision and drainage. The empirical antibiotics used were ceftriaxon 1 gm Q12h and metronidazole 500 mg Q8h, based on previous reports, to cover aerobic and anaerobic bacteria before the culture results were available [10,11].

### 2.1. Exclusion Criteria

Patients with mis-swallowing of a foreign body, severe cardiopulmonary diseases, previous head and neck tumor surgery, and previous radiotherapy over the head and neck region, as well as immunocompromised patients, were excluded. A total of 502 patients with DNI were enrolled in the study, among whom 30 patients had cooccurrence of DNI and AE at the time of diagnosis.

### 2.2. Data Collection

**Figure 2:**
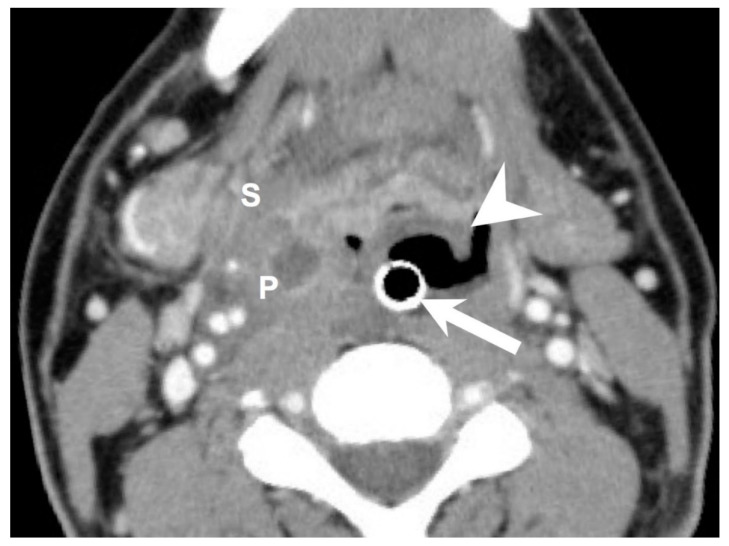
Axial CT view of an intubated patient with concurrent acute epiglottitis and multiple deep neck infections. Abscesses were detected in the parapharyngeal space and submandibular space. Arrow: endotracheal tube insertion; arrowhead: swollen and inflammatory epiglottis; P: parapharyngeal space; S: submandibular space. 300 × 300 DPI.

To investigate the risk factors associated with concurrent DNI and AE, we collected data on the patients’ gender, age, hospital stay, C-reactive protein (CRP) level, blood sugar level, diabetes mellitus (DM) status, performance of incision and drainage surgery, number of spaces affected by DNI, deep neck space involvement (Figure 2), presence of mediastinitis, tracheostomy, and species of pathogens involved.

### 2.3. Ethics Statement

This study was approved by the Institutional Review Board (IRB) of the Chang Gung Medical Foundation (IRB no. 202101961B0). The data were collected retrospectively and the patients were anonymized before data analysis. The IRB waived the need for informed consent.

### 2.4. Statistical Analysis

All data were analyzed using MedCalc software (ver. 18.6; MedCalc, Ostend, Belgium). As the Kolmogorov–Smirnov test showed that the data were not normally distributed, we used the chi-square test for categorical variables, Mann–Whitney U test for the comparison of continuous variables, and logistic regression analysis for univariate and multivariate analyses. A multivariate forward stepwise selection procedure was implemented, and all of the variables included in the univariate analysis were entered into the final multivariate model. In all analyses, *p* < 0.05 was taken to indicate statistical significance.

## 3. Results

Demographic and clinical data are shown in Table 1. A total of 502 patients with DNI, consisting of 325 men (64.74%) and 177 women (35.26%) with a mean age of 51.99 ± 19.04 years, were enrolled. The mean hospital stay was 10.05 ± 8.33 days. With regard to laboratory data, the mean CRP level was 148.54 ± 108.89 mg/L and the mean blood sugar level was 144.95 ± 73.73 mg/dL. A total of 207 (41.23%) patients had DM, and 236 (47.01%) patients underwent incisional and drainage surgery for DNI.

Among these patients, 196 (39.04%) had involvement of a single deep neck space, 146 (29.08%) had involvement of two spaces, and 160 (31.88%) had involvement of more than three spaces.

With regard to deep neck space involvement, 300 (59.76%) patients had parapharyngeal space involvement, 234 (46.61%) had submandibular space involvement, 164 (32.66%) patients had retropharyngeal space involvement, 106 (21.15%) had masticator space involvement, 85 (16.93%) patients had parotid space involvement, 42 (8.36%) had anterior cervical space involvement, 32 (6.37%) patients had carotid space involvement, 30 (5.97%) had visceral space involvement, 21 (4.18%) had perivertebral space involvement, and 12 (2.39%) had posterior cervical space involvement. Mediastinitis was found in 46 (9.16%) patients. Tracheostomy was performed in 68 (13.54%) patients. AE was found in 30 (5.97%) patients. Table 1 lists the pathogens cultured from these patients. No specific pathogens were cultured from 79 (15.73%) patients. 

Table 2 shows the results of a univariate analysis of variables for the 502 patients with DNI. The results show that parapharyngeal space involvement and submandibular space involvement were significant risk factors for AE (OR = 21.50, 95% CI: 2.905–158.7, *p* < 0.001 and OR = 2.064, 95% CI: 0.961–4.434, *p* < 0.001, respectively).

In Table 2, all factors were entered into a forward stepwise multivariate logistic regression model. Parapharyngeal space involvement (OR = 23.69, 95% CI: 3.187–175.4, *p* = 0.002) and submandibular space involvement (OR = 2.465, 95% CI: 1.131–5.375, *p* = 0.023) were significant independent risk factors for AE in patients with DNI.

As shown in Table 3, there were no significant differences in pathogens between the 30 patients with concurrent DNI and AE and the 472 patients with DNI alone (all *p* > 0.05). No specific pathogens were grown in blood cultures from three patients (10.00%) in the concurrent DNI and AE group and in 76 patients (15.25%) in the DNI alone group (*p* > 0.05).

In Table 4, there were no significant differences in therapeutic managements and hospital staying period between the 30 patients with concurrent DNI and AE and the 472 patients with DNI alone (all *p* > 0.05). 

## 4. Discussion 

In our study, involvement of the parapharyngeal space and involvement of the submandibular space were independent risk factors associated with concurrent AE and DNI. In addition, there were no differences in pathogens between the group with concurrent AE and DNI and the group with DNI alone.

The deep neck spaces lie within a complex framework formed by the cervical fascial planes [12]. As shown in Table 2, parapharyngeal space involvement and submandibular space involvement were independent risk factors for concurrent DNI and AE. The parapharyngeal space is a deep potential neck space shaped as an inverted pyramid, extending from the base of the skull to the hyoid bone [13]. The importance of the parapharyngeal space also lies in its connection with the other spaces of the neck. The masticator and parotid spaces are located laterally, the pharyngeal mucosal space is located medially, and the retropharyngeal space is located posteromedially [14]. The submandibular space is bounded anteriorly and laterally by the mandible, medially by the anterior belly of the digastric muscle, superiorly by the mylohyoid muscle, and inferiorly by the hyoid bone, while it also permits communication with the parapharyngeal space [15]. Therefore, infections can spread between the parapharyngeal space and submandibular space [16]. 

The epiglottis is formed of elastic cartilage [17]. Epiglottic inflammation can connect to the paraglottic and pre-epiglottic spaces and the parapharyngeal space [18]. Therefore, with the involvement of the parapharyngeal space and submandibular space, concurrent epiglottitis might occur, which can further lead to airway obstruction. In addition to concurrent DNI and AE, infection can be lethal in cases in which the DNI involves multiple spaces [1].

Proper protection of the airway with close surveillance of respiratory function and adequate infection control are important for the management of concurrent DNI and AE [19]. Airway specialists, such as otolaryngologists and anesthesiologists, should ideally evaluate the patient immediately to give sufficient time for preparation to secure the airway. In this cohort, tracheostomy was performed in 68 (13.54%) cases. The administration of effective broad-spectrum antibiotics against causative organisms is still important in the management of a severe infection. The involvement of multiple deep neck spaces was previously reported to be a risk factor for requiring tracheostomy [20]. 

Although many advances in diagnosis and treatment have been made, both DNI and AE can lead to rapid mortality if not managed appropriately. Patients with concurrent DNI and AE should be observed in a high acuity, closely monitored environment, such as the ICU, until the resolution of airway signs and symptoms [21].

Bacterial infection is the most common etiology of AE and DNI [7]. However, pathogens may vary geographically [22]. As shown in Table 3, there were no significant differences in pathogens between the 30 patients with concurrent DNI with AE and the 472 patients with DNI alone. In fact, DNI has a number of clinical presentations depending on the pathogenic organisms involved [23]. *Streptococcus constellatus* was the most commonly cultivated pathogen in the DNI patients regardless of whether they had concurrent AE (16.66%) or DNI alone (17.32%) (Table 3). This microorganism is a small catalase-negative coccus, which belongs to the Anginosus group, formerly known as *Streptococcus milleri* (along with *Streptococcus anginosus* and *Streptococcus intermedius*) [24]. *Streptococcus constellatus* infection was reported to be more common in patients aged between 35 and 54 years than in other age groups [25]. This microorganism behaves as a commensal organism of the oral cavity and oropharynx, but it can become invasive and pathogenic after mucosal disruption [23,26,27]. *Streptococcus constellatus* is known to be susceptible to β-lactam antibiotics, erythromycin, doxycycline, and vancomycin [28]. However, increases have been reported in resistance to penicillin and clindamycin [29]. The rate of specific pathogen non-growth in this cohort was 15.73% (79/502). However, blood culture is not a highly sensitive method for identifying pathogens, especially when antibiotics have already been administered [30].

In Table 4, there were no differences in therapeutic managements (tracheostomy, intubation, surgical drainage) or hospital staying period between the 30 patients with concurrent DNI and AE and the 472 patients with DNI alone. However, we consider concurrent AE and DNI is not simply a variant of DNI. We believe it is significant that DNI and AE are concurrent because coincidence of these two diseases makes airway protection more difficult. Therefore, these patients cause clinicians not only to pay more attention but also to make more effort to treat.

### Limitations of the Article

This study has some limitations. First, the retrospective nature of the study resulted in a certain attrition rate. In addition, most patients were male, which could have been due to selection bias.

## 5. Conclusions

Both DNI and AE are serious illnesses, and infections in critical spaces may cause the cooccurrence of these two diseases. Involvement of the parapharyngeal space and involvement of the submandibular space were independent risk factors for concurrent DNI and AE. There were no differences in pathogens, therapeutic managements, or hospital staying period between the concurrent DNI and AE group and the DNI alone group.

## Figures and Tables

**Table 1:** Clinicopathological characteristics of the 502 patients with deep neck infection.

Characteristics	N (%)
Gender	502 (100.00)
Male	325 (64.74)
Female	177 (35.26)
Age, years (SD)	51.99 ± 19.04
Hospital staying period, days (SD)	10.05 ± 8.33
CRP, mg/L (SD)	148.54 ± 108.89
Blood sugar, mg/dL (SD)	144.95 ± 73.73
Diabetes mellitus	207 (41.23)
Incision & drainage open surgery	236 (47.01)
Number of deep neck space involvement	
Single space	196 (39.04)
Double spaces	146 (29.08)
Multiple spaces, ≥3	160 (31.88)
Deep neck space involvement	
Parapharyngeal space	300 (59.76)
Submandibular space	234 (46.61)
Retropharyngeal space	164 (32.66)
Masticator space	106 (21.15)
Parotid space	85 (16.93)
Anterior cervical space	42 (8.36)
Carotid space	32 (6.37)
Visceral space	30 (5.97)
Perivertebral space	21 (4.18)
Posterior cervical space	12 (2.39)
Mediastinitis	46 (9.16)
Tracheostomy	68 (13.54)
Acute epiglottitis	30 (5.97)
Pathogens	
* Streptococcus constellatus*	87 (17.33)
* Parvimonas micra*	64 (12.47)
* Klebsiella pneumonia*	59 (11.75)
* Prevotella buccae*	56 (11.15)
* Prevotella intermedia*	53 (10.55)
* Streptococcus anginosus*	52 (10.35)
* Staphylococcus aureus*	25 (4.98)
* Staphylococcus epidemidis*	18 (3.58)
* Streptococcus salivarius*	16 (3.18)
* Streptococcus oralis*	12 (2.39)
* Eikenella corrodens*	10 (1.99)
* Gemella morbillorum*	10 (1.99)
* Slackia exigua*	9 (1.79)
* Salmonella enterica*	9 (1.79)
* Pseudomonas aeruginosa*	5 (0.99)
No growth	79 (15.73)

N = numbers; SD = standard deviation; CRP = C-reactive protein (normal range < 5 mg/L); blood sugar normal range: 70–100 mg/dL.

**Table 2:** Univariate and multivariate analyses of 30 cases of concurrent acute epiglottitis in 502 patients with deep neck infection.

Variable	Acute Epiglottitis		Univariate Analysis			Multivariate Analysis		
	Yes	No	OR	95% CI	*p* Value	OR	95% CI	*p* Value
Gender	30	472			0.342			
Male	17	308	0.696	0.473–2.110				
Female	13	164	1.000					
Age, years					0.442			
>50	18	249	1.344	0.470–2.122				
≤50	12	223	1.000					
CRP, mg/L (SD)	163.46 ± 114.14	147.59 ± 108.60	1.001	0.998–1.004	0.439			
Blood sugar, mg/dL (SD)	130.63 ± 25.98	145.86 ± 75.68	0.996	0.990–1.002	0.234			
Diabetes mellitus					0.809			
Yes	13	194	0.913	0.474–2.107				
No	17	278	1.000					
Multiple spaces, ≥3					0.287			
Yes	7	153	0.634	0.419–2.382				
No	23	319	1.000					
Parapharyngeal space					<0.001 *			0.002 *
Yes	29	271	21.50	2.905–158.7		23.69	3.187–175.4	
No	1	201	1.000			1.000		
Submandibular space					<0.001 *			0.023 *
Yes	19	215	2.064	0.961–4.434		2.465	1.131–5.375	
No	11	257	1.000			1.000		
Retropharyngeal space					0.160			
Yes	6	158	0.496	0.199–1.240				
No	24	314	1.000					
Masticator space					0.095			
Yes	3	103	0.398	0.118–1.338				
No	27	369	1.000					
Parotid space					0.086			
Yes	2	83	0.334	0.078–1.432				
No	28	389	1.000					
								
Anterior cervical space					0.250			
Yes	1	41	0.362	0.048–2.730				
No	29	431	1.000					
Carotid space					0.443			
Yes	1	31	0.490	0.064–3.722				
No	29	441	1.000					
Visceral space					0.123			
Yes	4	26	2.639	0.857–8.124				
No	26	446	1.000					
Perivertebral space					0.804			
Yes	1	20	0.779	0.101–6.012				
No	29	452	1.000					
Posterior cervical space					0.186			
Yes	2	10	3.300	0.689–15.78				
No	28	462	1.000					
Mediastinitis					0.871			
Yes	3	43	1.108	0.322–3.805				
No	27	429	1.000					

SD = standard deviation; OR = odds ratio; CI = confidence intervals; CRP = C-reactive protein; *, *p* < 0.05. Significant differences are shown in bold.

**Table 3:** Comparison of pathogens between 30 patients with concurrent acute epiglottitis and deep neck infection and 472 patients with deep neck infection alone.

Pathogens	Acute Epiglottitis, N (%)	Non-Acute Epiglottitis, N (%)	*p* Value
*Streptococcus constellatus*	5 (16.66)	82 (17.32)	1.000
*Parvimonas micra*	3 (10.00)	61 (12.92)	1.000
*Klebsiella pneumonia*	2 (6.66)	57 (12.07)	0.560
*Prevotella buccae*	3 (10.00)	53 (11.22)	1.000
*Prevotella intermedia*	3 (10.00)	50 (10.59)	1.000
*Streptococcus anginosus*	4 (13.33)	48 (10.16)	0.537
*Staphylococcus aureus*	3 (10.00)	22 (4.66)	0.181
*Staphylococcus epidemidis*	3 (10.00)	15 (3.17)	0.085
*Streptococcus salivarius*	2 (6.66)	14 (2.96)	0.246
*Streptococcus oralis*	2 (6.66)	10 (2.11)	0.157
*Eikenella corrodens*	2 (6.66)	8 (1.42)	0.115
*Gemella morbillorum*	1 (3.33)	9 (1.90)	0.463
*Slackia exigua*	2 (6.66)	7 (1.48)	0.095
*Salmonella enterica*	2 (6.66)	7 (1.48)	0.095
*Pseudomonas aeruginosa*	0 (0.00)	5 (1.05)	1.000
No growth	3 (10.00)	76 (16.10)	0.603

N = number.

**Table 4:** Comparison of managements and hospital staying period between 30 patients with concurrent acute epiglottitis and deep neck infection and 472 patients with deep neck infection alone.

Characteristics	Acute Epiglottitis, N (%)		Non-Acute Epiglottitis, N (%)		*p* Value
	Yes	No	Yes	No	
Tracheostomy	13 (10.00)	27 (90.00)	65 (13.77)	407 (86.23)	0.783
Intubation	16 (53.34)	14 (46.66)	226 (47.88)	246 (52.12)	0.578
I&D, open surgery	15 (50.00)	15 (50.00)	221 (46.82)	251 (53.18)	0.850
Hospital staying period, days (SD)	11.06 ± 6.13		9.98 ± 8.46		0.064

N = number; I&D = incision and drainage.

## Data Availability

All data generated or analyzed during this study are included in this published article. The data are available on request.

## Abbreviations

DNI: deep neck infection; AE: acute epiglottitis; US: ultrasonography; CT: computed tomography; CRP: C-reactive protein; DM: diabetes mellitus; OR: odds ratio.

## References

[1] Velhonoja J., Laaveri M., Soukka T., Irjala H., Kinnunen I. (2020). Deep neck space infections: An upward trend and changing characteristics. Eur. Arch. Otorhinolaryngol..

[2] Tapiovaara L., Back L., Aro K. (2017). Comparison of intubation and tracheotomy in patients with deep neck infection. Eur. Arch. Otorhinolaryngol..

[3] Rzepakowska A., Rytel A., Krawczyk P., Osuch-Wojcikiewicz E., Widlak I., Deja M., Niemczyk K. (2021). The Factors Contributing to Efficiency in Surgical Management of Purulent Infections of Deep Neck Spaces. Ear Nose Throat J..

[4] Boscolo-Rizzo P., Marchiori C., Montolli F., Vaglia A., Da Mosto M.C. (2006). Deep neck infections: A constant challenge. ORL.

[5] Prado-Calleros H.M., Jimenez-Fuentes E., Jimenez-Escobar I. (2016). Descending necrotizing mediastinitis: Systematic review on its treatment in the last 6 years, 75 years after its description. Head Neck.

[6] Aizawa N., Tsuchiya A., Takahashi S. (2013). Two cases of deep neck infection with esophageal perforation. J.-STAGE.

[7] Tsai Y.T., Huang E.I., Chang G.H., Tsai M.S., Hsu C.M., Yang Y.H., Lin M.H., Liu C.Y., Li H.Y. (2018). Risk of acute epiglottitis in patients with preexisting diabetes mellitus: A population-based case-control study. PLoS ONE.

[8] Komasawa N., Minami T. (2014). Difficult airway management in a patient with combined severe deep neck abscess and acute epiglottitis with abscess. J. Clin. Anesth..

[9] Ito K., Chitose H., Koganemaru M. (2011). Four cases of acute epiglottitis with a peritonsillar abscess. Auris Nasus Larynx.

[10] Yang S.W., Lee M.H., See L.C., Huang S.H., Chen T.M., Chen T.A. (2008). Deep neck abscess: An analysis of microbial etiology and the effectiveness of antibiotics. Infect. Drug Resist..

[11] Chen M.K., Wen Y.S., Chang C.C., Lee H.S., Huang M.T., Hsiao H.C. (2000). Deep neck infections in diabetic patients. Am. J. Otolaryngol..

[12] Vieira F., Allen S.M., Stocks R.M., Thompson J.W. (2008). Deep neck infection. Otolaryngol. Clin. N. Am..

[13] Unger J.M., Chintapalli K.N. (1983). Computed tomography of the parapharyngeal space. J. Comput. Assist. Tomogr..

[14] Shin J.H., Lee H.K., Kim S.Y., Choi C.G., Suh D.C. (2001). Imaging of parapharyngeal space lesions: Focus on the prestyloid compartment. Am. J. Roentgenol..

[15] Patel S., Bhatt A.A. (2018). Imaging of the sublingual and submandibular spaces. Insights Imaging.

[16] Huang C.M., Huang F.L., Chien Y.L., Chen P.Y. (2017). Deep neck infections in children. J. Microbiol. Immunol. Infect..

[17] Milroy C.M. (1992). Ossification of the epiglottis. J. Laryngol. Otol..

[18] Sideris G., Sapountzi M., Maragoudakis P., Delides A. (2020). Paraglottic and Pre-epiglottic Space Abscess in Adults: Report of Two Cases. Iran. J. Otorhinolaryngol..

[19] Pineau P.M., Gautier J., Pineau A., Emam N., Laccourreye L., Boucher S. (2021). Intubation decision criteria in adult epiglottitis. Eur. Ann. Otorhinolaryngol. Head Neck Dis..

[20] Chen S.L., Young C.K., Tsai T.Y., Chien H.T., Kang C.J., Liao C.T., Huang S.F. (2021). Factors Affecting the Necessity of Tracheostomy in Patients with Deep Neck Infection. Diagnostics.

[21] Dowdy R.A.E., Cornelius B.W. (2020). Medical Management of Epiglottitis. Anesth. Prog..

[22] Chen S.L., Young C.K., Liao C.T., Tsai T.Y., Kang C.J., Huang S.F. (2021). Parotid Space, a Different Space from Other Deep Neck Infection Spaces. Microorganisms.

[23] Han J.K., Kerschner J.E. (2001). Streptococcus milleri: An organism for head and neck infections and abscess. Arch. Otolaryngol. Head Neck Surg..

[24] Chrastek D., Hickman S., Sitaranjan D., Vokshi I., Kakisi O., Kadlec J., Bartosik W., Van Tornout F., Kouritas V. (2020). Streptococcus constellatus Causing Empyema and Sepsis, Necessitating Early Surgical Decortication. Case Rep. Infect. Dis..

[25] Jiang S., Li M., Fu T., Shan F., Jiang L., Shao Z. (2020). Clinical Characteristics of Infections Caused by *Streptococcus anginosus* Group. Sci. Rep..

[26] Gossling J. (1988). Occurrence and pathogenicity of the *Streptococcus milleri* group. Rev. Infect. Dis..

[27] Whiley R.A., Beighton D. (1991). Emended descriptions and recognition of *Streptococcus constellatus*, *Streptococcus intermedius*, and *Streptococcus anginosus* as distinct species. Int. J. Syst. Bacteriol..

[28] Nalmas S., Bishburg E., Chan T. (2007). *Streptococcus constellatus* and *Prevotella bivia* penile abscess. Sci. World J..

[29] Tracy M., Wanahita A., Shuhatovich Y., Goldsmith E.A., Clarridge J.E., Musher D.M. (2001). Antibiotic susceptibilities of genetically characterized *Streptococcus milleri* group strains. Antimicrob. Agents Chemother..

[30] Guardiani E., Bliss M., Harley E. (2010). Supraglottitis in the era following widespread immunization against *Haemophilus influenzae* type B: Evolving principles in diagnosis and management. Laryngoscope.

